# Tumor microenvironment-associated lactate metabolism regulates the prognosis and precise checkpoint immunotherapy outcomes of patients with lung adenocarcinoma

**DOI:** 10.1186/s40001-022-00895-6

**Published:** 2022-11-21

**Authors:** Song Qiu, Ying Wang, Hui Rao, Qiuyang Que, Yanyang Wu, Rui Zhu, Xiaofei Feng, Jun Chi, Weiling Lai, Yihang Sun, Qi Xiao, Huaqiu Shi, Yi Xiang

**Affiliations:** 1 Department of Oncology, The First Affiliated Hospital, Gannan Medical University, No 23, Qingnian Road, Ganzhou, China; 2 School of Pharmaceutical Sciences, Southern Medical University, Guangzhou, China; 3 Jiangkou Town Central Health Center, Ganxian District, Ganzhou, China

**Keywords:** Lactate metabolism, Gene signature, Risk score, Prognosis, Immunotherapy benefit, Lung adenocarcinoma

## Abstract

**Background:**

Despite the wide clinical application of checkpoint inhibitor immunotherapy in lung adenocarcinoma, its limited benefit to patients remains puzzling to researchers. One of the mechanisms of immunotherapy resistance may be the dysregulation of lactate metabolism in the immunosuppressive tumor microenvironment (TME), which can inhibit dendritic cell maturation and prevent T-cell invasion into tumors. However, the key genes related to lactate metabolism and their influence on the immunotherapeutic effects in lung adenocarcinoma have not yet been investigated in depth.

**Methods:**

In this study, we first surveyed the dysregulated expression of genes related to lactate metabolism in lung adenocarcinoma and then characterized their biological functions. Using machine learning methods, we constructed a lactate-associated gene signature in The Cancer Genome Atlas cohort and validated its effectiveness in predicting the prognosis and immunotherapy outcomes of patients in the Gene Expression Omnibus cohorts.

**Results:**

A 7-gene signature based on the metabolomics related to lactate metabolism was found to be associated with multiple important clinical features of cancer and was an independent prognostic factor.

**Conclusions:**

These results suggest that rather than being simply a metabolic byproduct of glycolysis, lactate in the TME can affect immunotherapy outcomes. Therefore, the mechanism underlying this effect of lactate is worthy of further study.

**Supplementary Information:**

The online version contains supplementary material available at 10.1186/s40001-022-00895-6.

## Introduction

An important physiological function of glucose is to provide energy for cell survival. In the human body, glucose is metabolized through three main routes: anaerobic glycolysis, the pentose phosphate pathway, and mitochondrial oxidative phosphorylation [1, 2]. However, metabolic reprogramming is common in tumorigenesis and cancer development, endowing the tumor cells with multiple competitive advantages for survival and progression [3–5]. One such metabolic change is the increase in lactate production due to the Warburg effect of aerobic glycolysis [6–8]. Lactate metabolism in tumor cells is significantly different from that in normal cells [9]. Unlike normal cells that gain energy by metabolizing glucose to pyruvate, which is then transported into the mitochondria for full oxidative phosphorylation, tumor cells rely mainly on aerobic glycolysis for energy and producing precursors for protein, lipid, and nucleotide synthesis [4, 10, 11]. In tumor cells, glucose is first catalyzed to pyruvate by enzymes such as hexokinase, 6-phosphofructokinase-1, and pyruvate kinase [12]. Finally, the high expression of lactate dehydrogenase (LDH) and pyruvate dehydrogenase kinase in tumor tissue promotes the reduction of pyruvate to lactate [13].

This highly efficient aerobic glycolysis can produce a large amount of lactate, which can then be secreted into the extracellular space to acidify the tumor microenvironment (TME) [14, 15]. In the past, lactate was considered to be only metabolic waste as a byproduct of glycolysis. However, an increasing number of studies have found that lactate can activate many important signaling pathways in tumor cells to promote the survival, invasion, immune escape, metastasis, and angiogenesis of multiple types of cancer [16–20]. Baumann et al. [21] demonstrated that lactate could induce the upregulated expression of transforming growth factor-beta 2 (TGFβ2) to stimulate tumor invasion and metastasis. Other studies have shown that lactate acts as an immunosuppressive molecule by promoting the survival of regulatory T lymphocytes (Tregs) [19, 22, 23], suppressing the production of interferon-gamma (IFN-γ) in cytotoxic T cells [24, 25], inhibiting the toxicity and cytolytic functions of natural killer cells [26, 27], and facilitating M2 polarization of tumor-associated macrophages via the ERK/STAT3 signaling pathway [16, 28]. Additionally, the abnormal metabolism and excretion of lactate can suppress the degradation of hypoxia-inducible factor 1-alpha (HIF-1α) [29] and increase the production of vascular endothelial growth factor [30] and fibroblast growth factor [31] in endothelial cells, thereby promoting angiogenesis in the TME [20, 32, 33]. Therefore, more detailed studies on lactate metabolism and the key enzymes involved in the process may provide new insights into tumor pathogenesis as well as potential therapeutic targets.

To date, the effects of the dysregulation of lactate metabolism and related genes in the TME on the therapeutic outcomes and prognosis of cancer patients have not been studied. Moreover, there is a lack of research on the prognostic value of lactate metabolism in lung adenocarcinoma. Therefore, the objectives of this study were to identify the genes associated with lactate metabolism in lung adenocarcinoma and determine their expression characteristics and biological significance. To this end, data were downloaded from The Cancer Gene Atlas (TCGA) and the Gene Expression Onmibus (GEO) databases to identify the lactate metabolism-related genes in lung adenocarcinoma. Then, using machine learning tools, we constructed a prognostic risk score model based on lactate metabolism-associated genes screened from the TCGA dataset and evaluated its predictive effectiveness on the GEO patient cohorts. The 7-gene signature was found to be associated with multiple important clinical features of cancer. These results suggest that rather than being simply a metabolic byproduct of glycolysis, lactate in the TME can affect immunotherapy outcomes. Further elucidation of the mechanism underlying this lactate effect could provide a practical reference for the formulation of individualized treatment strategies.

## Materials and methods

### Data acquisition and pretreatment

The lung adenocarcinoma samples of The Cancer Genome Atlas (TCGA) database [34] downloaded from Xena (https://tcga-xena-hub.s3.us-east-1.amazonaws.com/download/TCGA.LUAD.sampleMap%2FHiSeqV2.gz) were used as the training set. GSE26939, a lung adenocarcinoma chipset [35], was downloaded from GEO database (https://www.ncbi.nlm.nih.gov/geo/query/acc.cgi?acc=GSE26939) for verification (shown in Table 1). We converted the gene expression data using log2-transformed quantile-normalized signal intensity. When there are multiple probes corresponding to the same gene, we take the median as the expression value. And then each probe was converted to corresponding gene symbol according to the labeling information.

**Table 1:** The expression profile dataset in lung adenocarcinoma downloaded from online databases

Dataset ID	Platform	Tumor	Normal
TCGA-LUAD	Illumina	517	59
GSE26939	GPL9053	116	0
GSE190266	GPL24676	70	0
GSE135222	GPL16791	27	0
GSE136961	GPL24014	21	0
GSE81089	GPL16791	198	22
GSE101929	GPL570	33	33

### Identification of lactate metabolism genes

Lactic acid, and lactate were used as the keyword to search for genes related to lactate metabolism in the GSEA database (http://www.gsea-msigdb.org/gsea/login.jsp). Meanwhile, lactate was used as the keyword on Genecard database (https://www.genecards.org/), and genes with score > 20 were selected as supplementary gene sets. By taking the intersection of different gene sets, we finally obtained 26 candidate genes related to lactate metabolism (the gene symbols and NCBI ID are listed in Additional file 1: Table S1): GOBP_LACTATE_METABOLIC_PROCESS; GOBP_LACTATE_TRANSMEMBRANE_TRANSPORT; GOMF_LACTATE_DEHYDROGENASE_ACTIVITY; GOMF_LACTATE_TRANSMEMBRANE_TRANSPORTER_ACTIVITY.

### Differential expression analysis

We then screened the differentially expressed genes (DEGs) of lung adenocarcinoma samples in the TCGA cohorts using R-package DESeq2. By using the Benjamini–Hochberg procedure to control the false discovery rate (FDR), 5530 DEGs were identified with a strict cut-off of *P* < 0.01 and an FDR of less than 0.05. And then 9 genes were screened out after the intersection of the 5530 DEGs with the candidate genes related to lactate metabolism.

### Machine learning of prognostic gene signatures based on lactate metabolism

We used R-package *GLMNET* [36] to perform Least Absolute Shrinkage and Selection Operator (LASSO) regression analysis [37] based on the expression matrix of lactate metabolism-related genes from the above univariate Cox regression, and found that the model has the highest accuracy when the degree of freedom (gene number) was 11 (Fig. 4A, B). Finally, the signature of 7 lactate metabolism-related genes was selected to assemble a prognostic risk score model (Fig. 4C). The lactate-related risk score of each patient was calculated by the formula: riskScore = (−0.06275159 *ACTN3 expression value −0.01796317 *LDHD expression value + 0.01581625 *LDHA expression value + 0.01821132 *SLC16A3 expression value + 0.04737492 *SLC16A1 expression value + 0.05394477 *HAGH expression value + 0.05710535 *DARS2 expression value).

### Immunotherapy cohorts and therapeutic benefit evaluation

In order to investigate the correlation between the 7-gene signature and clinical response, the sequencing and clinical data of patients with lung adenocarcinoma who received anti-PD-(L)1 immunotherapy in six different datasets [GSE190266 (*n* = 70) [38], GSE135222 (*n* = 27) [39], GSE136961 (*n* = 21) [40], GSE81089 (*n* = 198) [41], GSE101929 (*n* = 33) [42] and the MSS Mixed Solid Tumors cohort, from Dana-Farber Cancer Institute, Boston, USA (*n* = 47) [43]) were downloaded from GEO database and cBioPortal database [44]. We next explored the potential effect of lactate metabolism on anti-PD-(L)1 immunotherapy in patients based on the short-term and long-term analysis. The short-term analysis focused on the tumor objective response, while the long-term analysis focused on the progression-free survival (PFS) and overall survival (OS) of patients after receiving treatments.

### Statistical analysis

SPSS version 23.0 software (SPSS, Chicago, IL, USA) or R software (version 4.0.3) was used for statistical analysis. Chi-squared test was used for the clinicopathological parameters among different treatment groups. The log-rank test was used for the Kaplan–Meier survival of each group. Univariate and multivariate analysis were presented through Cox regressions. The reported results covered hazard ratios (HR) and 95% confidence intervals (CI). Curve analysis of the receiver operating characteristic (ROC) was applied for evaluating the predictive performance.

## Results

### Dysregulated expression and biological characterization of genes related to lactate metabolism in lung adenocarcinoma

**Fig. 1:**
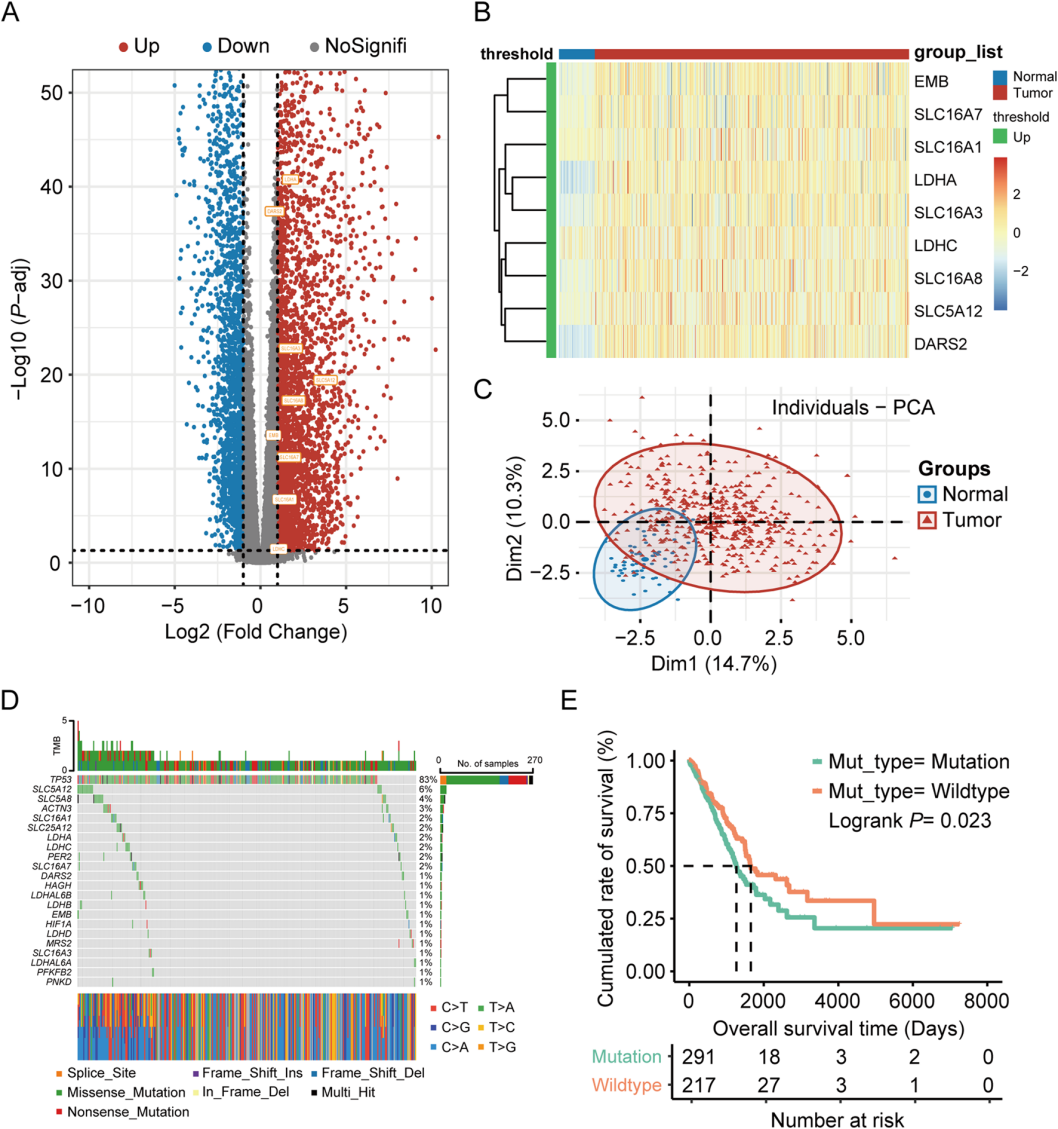
Dysregulated expression and biological characterizations of lactate-related genes in lung adenocarcinoma. **A** Volcano plot of the differential expressions of 5530 DEGs in TCGA cohort. **B** Heatmap of the differential expressions of DEGs related to lactate metabolism. **C** Sample dimensionality reduction by using principal component analysis (PCA) demonstrated the reliable identification value of these 9 lactate-related genes. **D** Waterfall-plot of single nucleotide variation of the 9 lactate-related genes. **E** The OS curves of patients in lactate-related gene mutation group and wildtype group

First, 5530 DEGs between the 517 cancer samples and 59 normal tissue samples from the TCGA database were extracted using the DESeq2 package. After intersection of these 5530 DEGs with the genes related to lactate metabolism (identified from the GSEA and GeneCards platforms), nine genes were screened for further study (Fig. 1A). The heatmap in Fig. 1B shows the differential expression of these nine genes in the TCGA cohort. To determine whether these lactate metabolism-related genes can distinguish cancer patients from healthy individuals, the sample dimensions of the genes were reduced using principal component analysis. As shown in Fig. 1C, these nine genes had reliable identification value.

Next, on the basis of the mutant MAF file from the TCGA database, a waterfall plot of single nucleotide variations of these nine genes was drawn using maftools in the R package (Fig. 1D) [45]. Subsequently, these genes were divided into mutation and wild-type groups for survival analysis. The results obtained using survminer in the R package demonstrated that the OS of the wild-type group was considerably longer than that of the mutation group (Fig. 1E).

### Copy number variations of genes related to lactate metabolism in lung adenocarcinoma

**Fig. 2:**
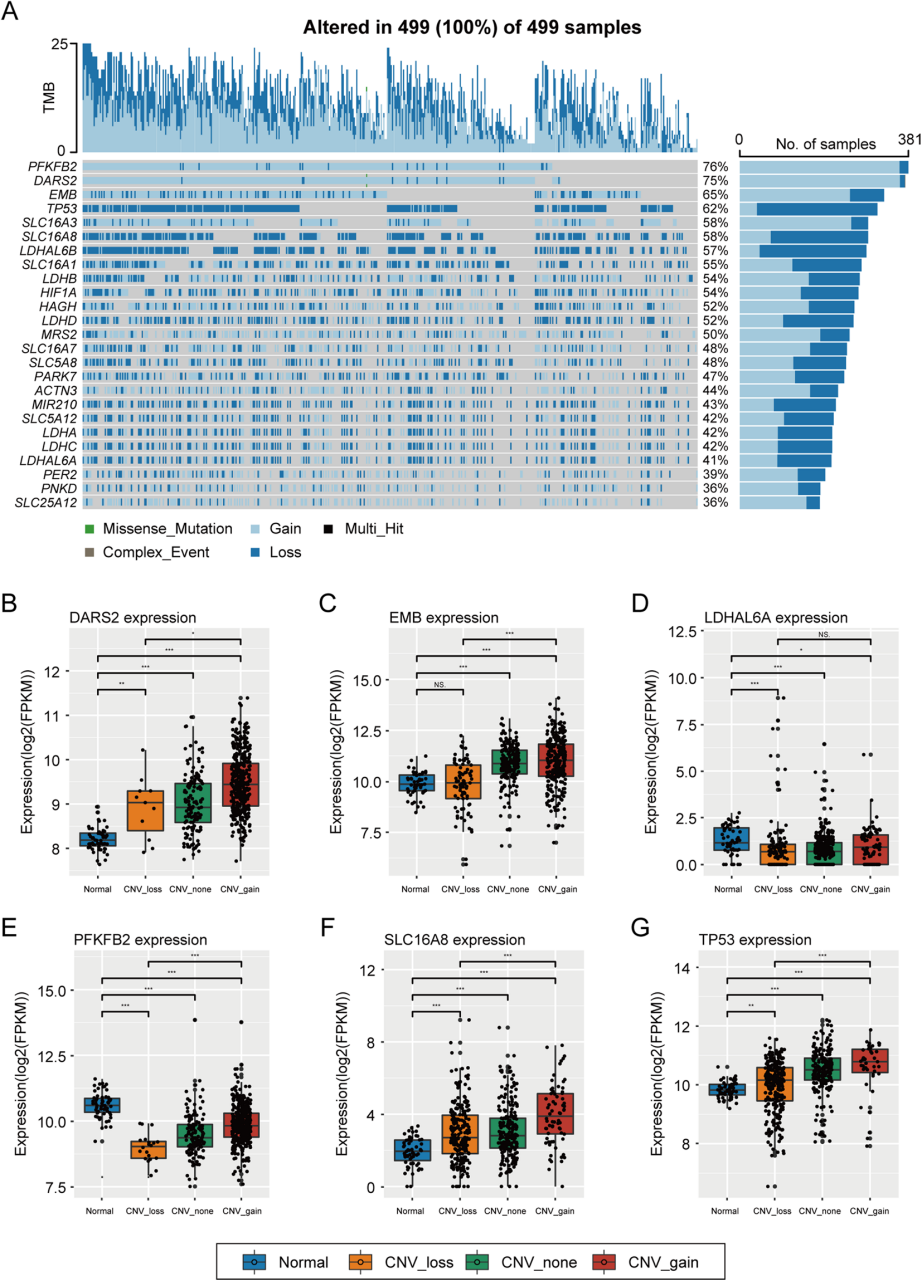
Copy number variations (CNVs) of lactate-related genes in lung adenocarcinoma. **A** Waterfall-plot of the CNVs of these lactate-related genes. **B**–**G** Box plots showed the differential expression levels of the top 6 lactate-related genes with the most CNVs. *P < 0.05; **P < 0.01; ***P < 0.001

We extracted the CNVs of all lactate metabolism-related genes to evaluate their copy status (Fig. 2A) and then selected the top six genes with the most CNVs. As illustrated by the box plots (Fig. 2B–G), the following six genes were significantly differentially expressed between the normal and cancerous tissues: aspartyl-tRNA synthetase, mitochondrial (*DARS2*), embigin (*EMB*), lactate dehydrogenase A like 6A (*LDHAL6A*), 6-phosphofructo-2-kinase/fructose-2,6-biphosphatase 2 (*PFKFB2*), solute carrier family 16 member 8 (*SLC16A8*), and tumor protein P53 (*TP53*).

### Prognostic performance of the lactate metabolism-related genes in lung adenocarcinoma

**Fig. 3:**
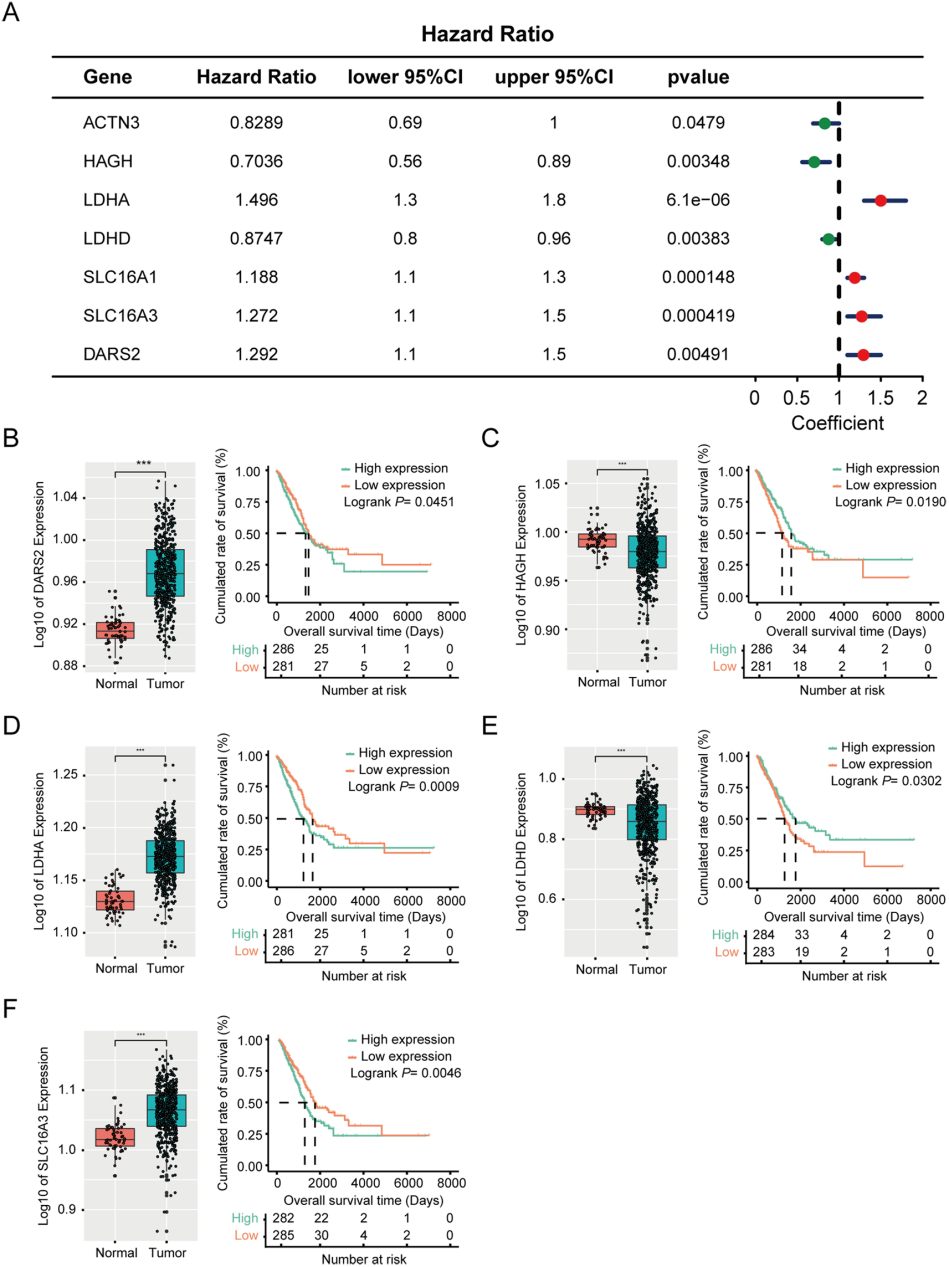
Prognostic performance of lactate-related genes in lung adenocarcinoma. **A** Forest-plot showed the lactate-related genes associated with the prognosis of patients with lung adenocarcinoma analyzed by univariate Cox regression. **B**–**F** Box plot and Kaplan–Meier curve exhibited the differential expressions and prognostic performance of the top 5 significant lactate-related genes. *P < 0.05; **P < 0.01; ***P < 0.001

Using the forestplot package, univariate Cox regression analysis was performed to evaluate the prognostic performance of the lactate metabolism-related genes. The forest plot in Fig. 3A shows the genes associated with patient prognosis. The samples were divided into high and low expression groups according to the median expression level. The box plot and Kaplan–Meier curve showed the following top five significant prognostic genes to be closely related to patient survival: *DARS2*, hydroxyacyl glutathione hydrolase (*HAGH*), lactate dehydrogenase A (*LDHA*), lactate dehydrogenase D (*LDHD*), and *SLC16A3* (Fig. 3B–F).

### Prognostic signature based on lactate metabolism-related genes screened from the TCGA cohort

**Fig. 4:**
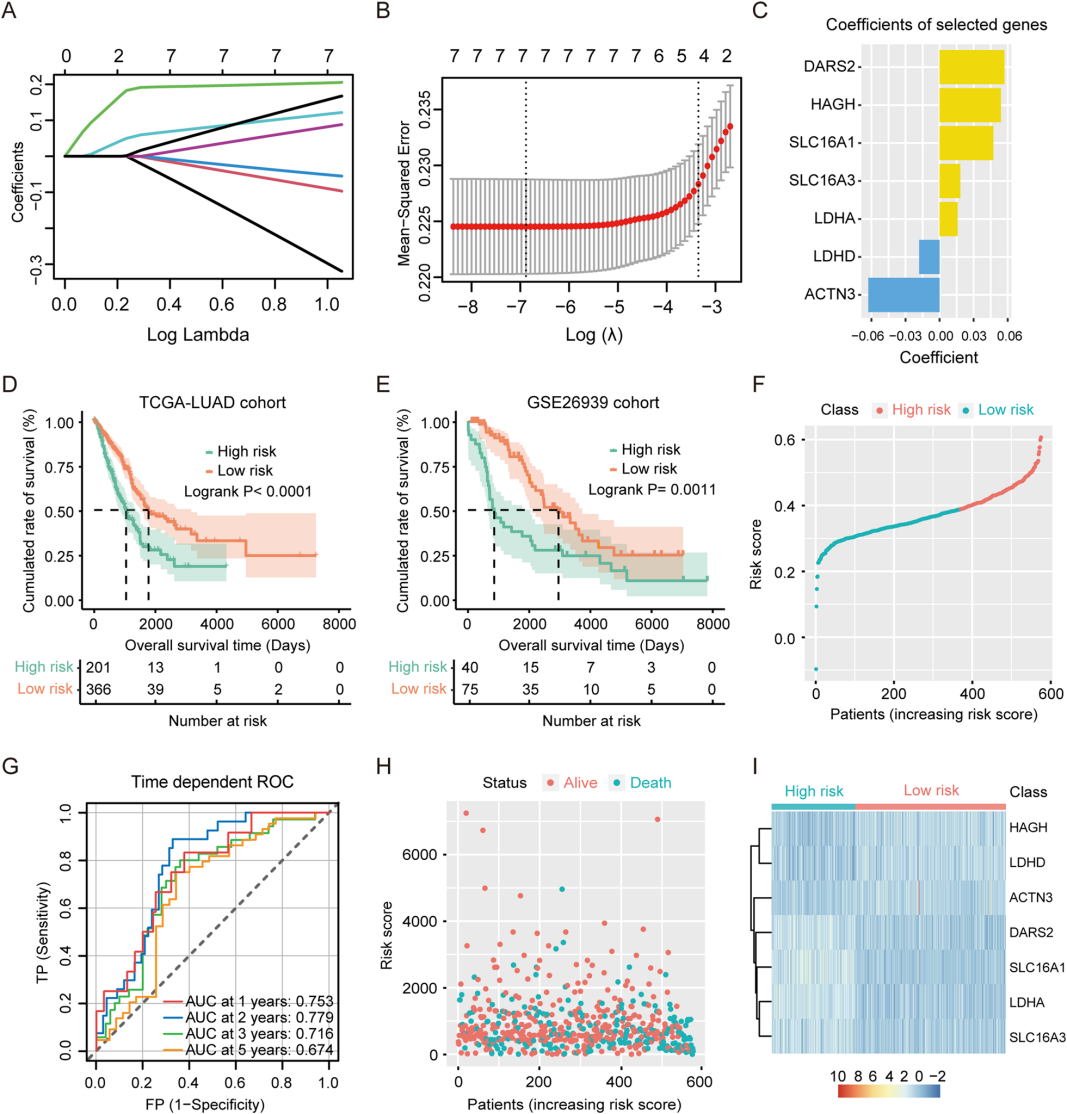
Machine learning of prognostic gene signatures based on lactate metabolism. **A** Dynamic process of variable screening by Least Absolute Shrinkage and Selection Operator (LASSO) regression analysis. **B**, **C** The cross-validation results of LASSO regression (**B**) and coefficients of selected genes (**C**). **D**, **E** The OS curves of the lactate-related gene signatures in TCGA-LUAD samples (**D**) and GSE26939 samples (**E**). **F** Patients with lung adenocarcinoma were divided into high-risk and low-risk groups, taken the best cutoff value as the threshold. **G** The receiver operating characteristic (ROC) curves for 1-year, 2-year, 3-year and 5-year OS. **H** Scatter-plot showed survival time of patients in high-risk and low-risk groups. **I** Heatmap of the differential expressions of the 7 lactate-related genes selected to construct a prognostic gene signature in lung adenocarcinoma

By constructing a gene signature of seven lactate metabolism-related genes that were screened using machine learning tools and LASSO regression analysis [37] (Fig. 4A–C), we were able to explore the effect of dysregulated lactate metabolism on patient survival. Patients with lung adenocarcinoma were divided into high- and low-risk groups (with the best cutoff value taken as the threshold) to test the prognostic performance of the constructed gene signature on TCGA-LUAD (training cohort) and GSE26939 (validation cohort) samples (Fig. 4D–F). The areas under the ROC curves (AUCs) for 1-, 2-, 3-, and 5-year OS were 0.753, 0.779, 0.716, and 0.674, respectively (Fig. 4G). The scatter plot in Fig. 4H shows the survival time of patients in the high- and low-risk groups, whereas the heatmap in Fig. 4I displays the differential expression levels of the seven genes selected to construct the gene signature. Additionally, a multivariate Cox analysis (Table 2), performed using the coxph() function of survival in the R package, revealed the 7-gene signature to be an independent prognostic factor. These results demonstrated that this lactate metabolism-related gene signature could effectively distinguish the prognosis of patients with lung adenocarcinoma.

**Table 2:** Multivariate Cox regression analysis for survival in patients with lung adenocarcinoma

Variables	Multivariate analysis			*P*-value
	Hazard ratio	Lower 95%CI	Upper 95%CI	
Lactate-related risk score	340.00	40.00	2900.00	< 0.0001***
Age (≥ 60 vs. < 60)	1.20	0.86	1.60	0.30
Gender (male vs. female)	0.98	0.74	1.30	0.87
Pathological stage (III–IV vs. I–II)	2.10	1.00	4.10	0.036*
Distant metastasis (M1 vs. M0)	0.82	0.37	1.80	0.62
Lymph node metastasis (N2-3 vs. N0-1)	0.92	0.47	1.80	0.80
Tumor invasion (T1-2 vs. T3-4)	0.72	0.11	4.60	0.73
Previous radiation therapy (yes vs. no)	1.50	1.10	2.20	0.02

*CI* confidence interval

*P < 0.05; **P < 0.01; ***P < 0.001

### Performance of the lactate metabolism-related gene signature in predicting PD-1 immunotherapy efficacy in patients with lung adenocarcinoma

**Fig. 5:**
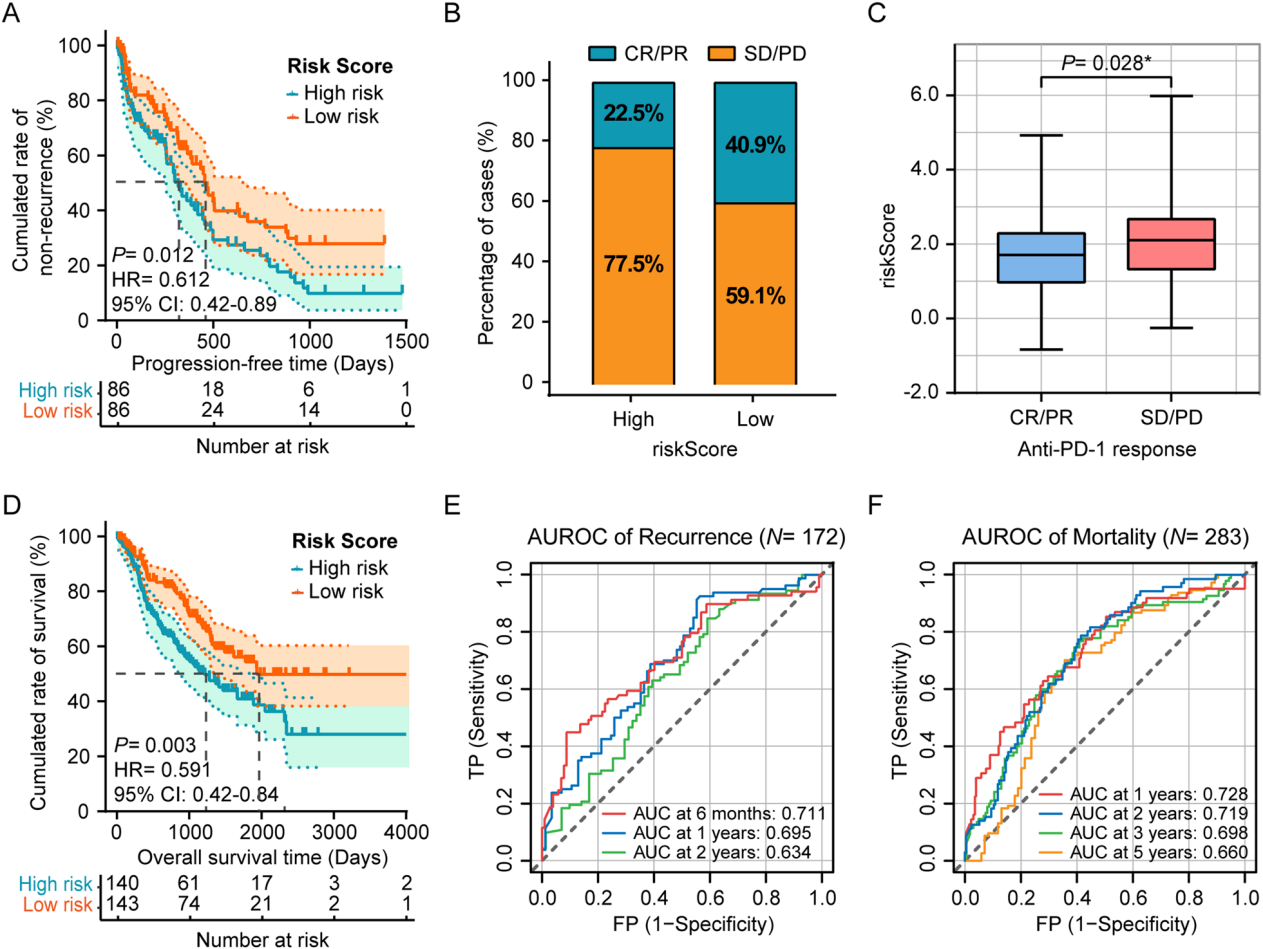
Efficacy prediction of PD-1 immunotherapy based on lactate metabolism in patients with lung adenocarcinoma. **A** Kaplan–Meier analysis demonstrated that the PFS of patients in the high-risk group were significantly shorter than those of the low-risk group. **B** The ORR of patients in high-risk and low-risk groups. **C** Box plot of the risk scores for different anti-tumor treatment responses. **D** The OS of patients in the high-risk and low-risk groups. **E**, **F** Time-dependent ROC curves for recurrence and mortality of patients at different follow-up times. *P < 0.05

Based on our short- and long-term analyses of six datasets of lung adenocarcinoma immunotherapies downloaded from the GEO database, we found that lactate metabolism may play a potential role in promoting the therapeutic effect of anti-PD-1 monoclonal antibodies. As shown in Fig. 5A, D, the PFS and OS of patients with high risk scores were significantly shorter than those of patients with low risk scores (*P* = 0.012 and *P* = 0.003, respectively). The overall response rates (ORRs) of the high- and low-risk groups were 22.5% and 40.9%, respectively (Fig. 5B). Patients who achieved an objective response also had a lower risk score than those who did not respond to treatment (*P* = 0.028, Fig. 5C). Ultimately, the ROC curves and the calculated AUCs (Fig. 5E, F) indicate that the constructed gene signature is a promising tool for predicting mortality and recurrence in lung adenocarcinoma patients who received anti-PD-1 immunotherapy.

### Correlation of the gene signature with multiple important clinical features of cancer

**Fig. 6:**
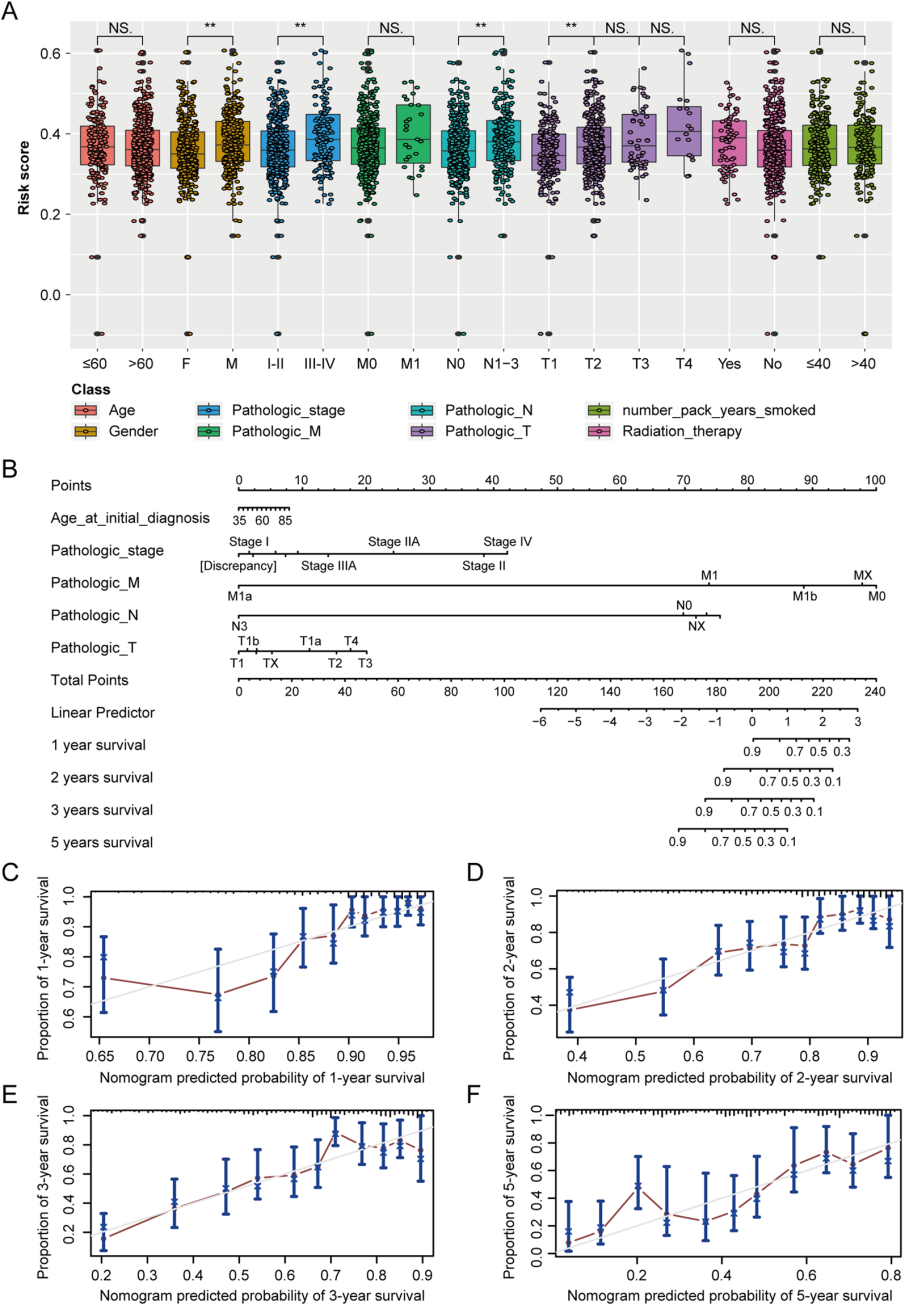
The lactate-related gene signature is associated with multiple important clinical features of cancer. **A** Box plots exhibited the correlations between the lactate-related gene signatures and various clinical features in lung adenocarcinoma. **B** The nomograms constructed based on the characteristics for guiding clinical practice. **C**–**F** The prediction outcomes of nomograms for 1-year, 2-year, 3-year and 5-year survival

Figure 6A shows the correlations between the lactate metabolism-related gene signature and common clinical characteristics of patients with lung adenocarcinoma, such as age, sex, TNM stage, and prior radiation therapy. Furthermore, we used the RMSE function in the R package to construct nomograms for guiding clinical practice [46] according to these clinical characteristics (Fig. 6B). The prediction outcomes for 1-, 2-, 3-, and 5-year survival are presented in Fig. 6C–F.

### Relationship between lactate metabolism and the tumor immune microenvironment

**Fig. 7:**
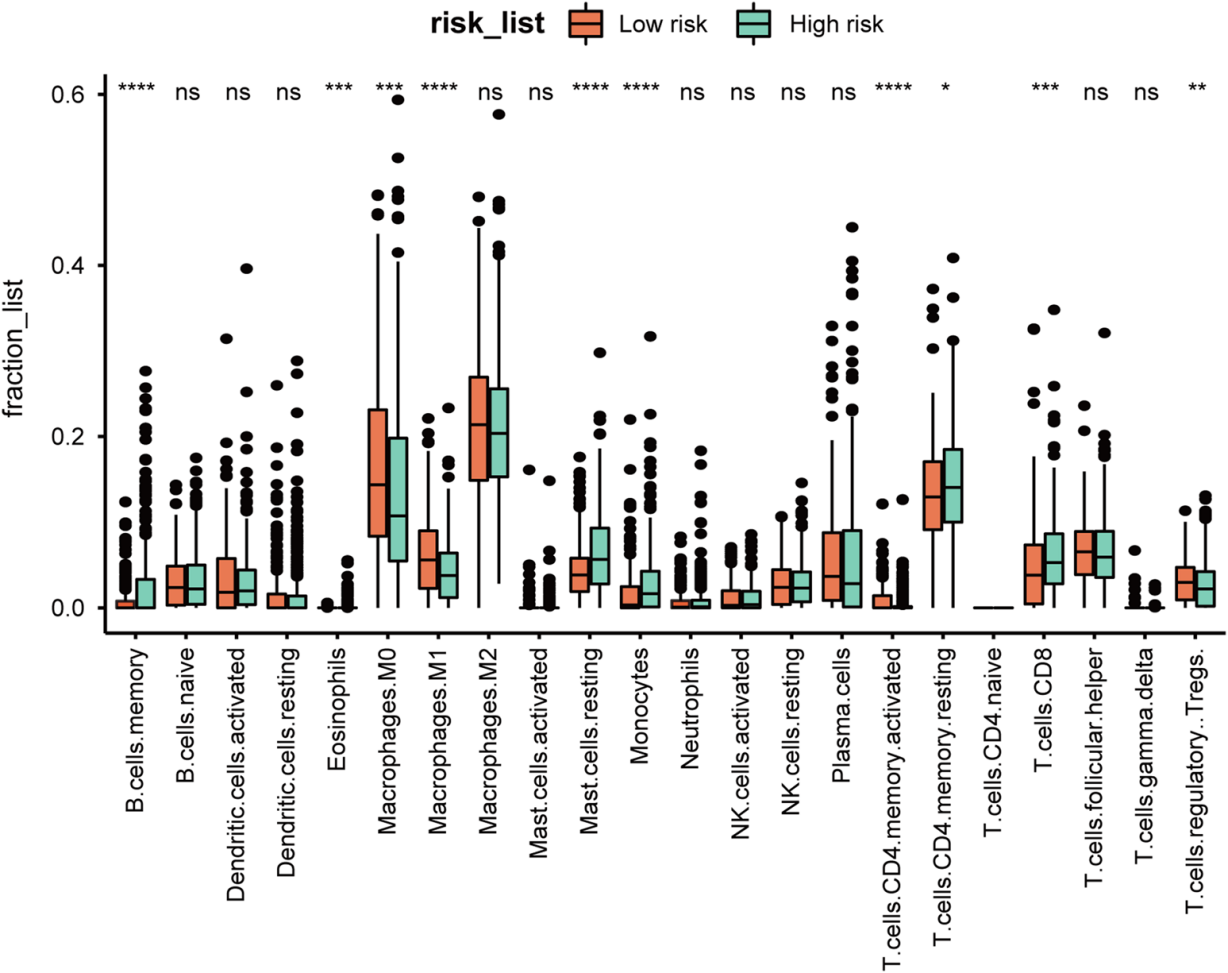
The infiltration proportion of various types of immune cells between high-risk and low-risk groups. *P < 0.05; **P < 0.01; ***P < 0.001

**Fig. 8:**
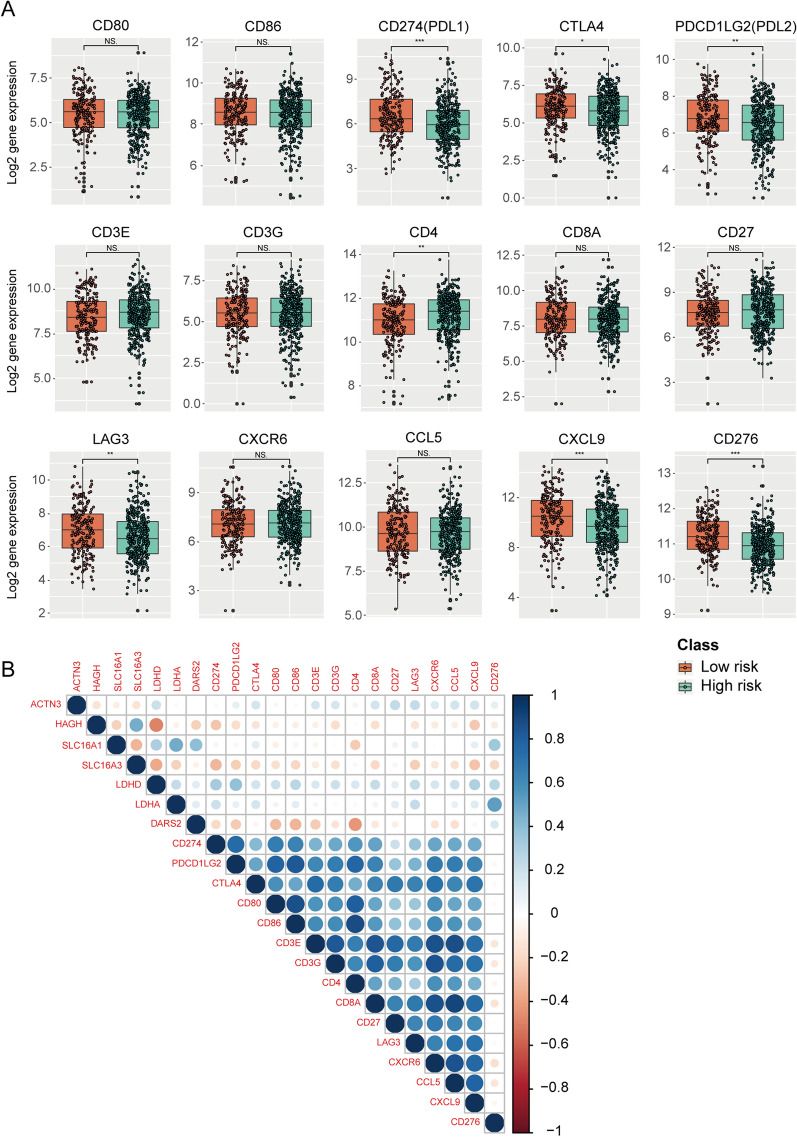
The relationship between lactate metabolism-related genes and immune checkpoint genes. **A** Box plots of the differential expression levels of several representative immune checkpoint genes between high-risk and low-risk groups. **B** Correlation analysis between genes in the signature and immune checkpoint genes (color of dots represents correlation coefficient). *P < 0.05; **P < 0.01; ***P < 0.001

We used CIBERSORT (https://cibersortx.stanford.edu/runcibersortx.php) [47] to calculate the proportion of each type of immune cell in the samples to assess the association between lactate metabolism and tumor immune microenvironment. The box plot in Fig. 7 shows the proportions of various infiltrating immune cells between the high and low risk-score groups. The differential expression of several immune checkpoint genes in each group was analyzed using ggplot2 in the R package (Fig. 8A). And the correlation coefficients between genes in our signature and immune checkpoint genes are exhibited in Fig. 8B. Finally, the gene sets of 50 cancer hallmark pathways were used to calculate the GSVA scores for the expression matrix. Spearman’s correlation analysis was performed to determine the relationships between the GSVA scores and the risk score of these samples calculated with the constructed gene signature. The correlations between the gene signature and the 50 cancer hallmark pathways are presented in Additional file 2: Fig. S1.

## Discussion

Increasing lines of evidence have shown that the occurrence, development, and prognosis of multiple cancer types are closely related to the TME [3, 48, 49]. As an important stress metabolite, lactate is produced through aerobic glycolysis in tumor cells, where its accumulated level could affect the expression of the cancerous cells as well as T cells, dendritic cells, and macrophages in the TME, thereby triggering intracellular signals [22, 23, 29, 50]. Lactate also has different effects on the biological processes of tumors, including their proliferation and migration, immune escape, and prognosis, as well as on the curative effects of various treatments. It was shown in a previous study that an overflow of proton-coupled lactate in cancerous or stromal cells could promote tumor progression by regulating various processes in the TME (including cell invasion, angiogenesis, survival signaling, metastasis development, and immune surveillance avoidance) [51]. Simultaneously, the acidic microenvironment formed by a large amount of lactate accumulation is conducive to tumor cell metastasis and angiogenesis [32, 52].

Many studies have pointed out that the lactate content in the TME is negatively correlated with the tumor survival rate [53, 54]. Thus, research on abnormal lactate metabolism has long-term prospects. Most of earlier studies about lactate metabolism genes either focus on the overall survival of patients with various cancer types, or explore their potential relationship with tumor immune microenvironment through evaluating the correlation with the expression of common immune biomarkers. However, the identification and performance of lactate metabolism-related gene signatures in predicting the clinical response and long-term benefits for patients receiving checkpoint immunotherapy is still not studied in depth. In this study, patients were stratified into two clusters with the median risk score as the cut-off point. We identified and validated the gene signature based on lactate metabolism in predicting immunotherapeutic response in lung adenocarcinoma. These results suggest that the lactate in TME can affect immunotherapy outcomes, rather than simply being a metabolic byproduct of glycolysis. The underlying mechanism needs further research and might provide future directions for formulating individualized treatment strategy (Additional file 3).

Machine learning algorithm is an intelligent tool that explores and simulates the law of human intelligent activities based on computer technology. Recently, as high-throughput sequencing has evolved, machine learning algorithms are gaining traction in medical research [55]. Stratifying tumor patients by bioinformatics and machine learning approaches to explore new biomarkers has proven to be reliable and useful. At present, there are few reports on the influence of dysregulated lactate metabolism on the immunotherapy response in lung cancer using these algorithms. LASSO analysis involved in our study is a powerful regression machine learning algorithm for processing high-dimensional data and feature selection. And the prognostic gene signature screened and developed by LASSO regression had been further evaluated by ROC analysis. In general, the combination of machine algorithm and clinical medicine may become a trend in future clinical research. With its high accuracy and operability, these algorithms can greatly improve the efficiency of clinical practice, and provide a new perspective for diseases research.

The gene signature constructed in this study was based on seven differentially expressed lactate metabolism-related genes screened from TCGA cancer samples: actinin alpha 3 (*ACTN3*), *SLC16A1*, *SLC16A3*, *LDHA*, *LDHD*, *HAGH*, and *DARS2*. ACTN3, a structural protein of the Z-line of fast skeletal muscle fibers [56], maintains the orderly arrangement and normal contraction of muscle fibers by cross-linking with fine muscle filaments. SLC16A1 and SLC16A3 belong to the solute carrier protein 16A family, also known as the monocarboxylic acid transporter family [57]. They mediate the transmembrane transport of monocarboxylic acids dominated by lactate and short-chain fatty acids. Tumor cells can promote lactate production through the enzyme LDHA, thereby destroying tumor-infiltrating T cells as well as IFN-γ in natural killer cells and other cytokines to promote epithelial–mesenchymal transformation, angiogenesis, and invasion [26]. With regard to the human *HAGH* gene, its extended transcripts have been shown to encode both cytosolic and mitochondrial isoforms of glyoxalase II [58]. As for the *DARS2* gene, its abnormal expression is reportedly involved in the occurrence and development of primary liver carcinoma, with the expression level being closely related to the tumor size, stage, and prognosis [59].

Notably, the present study has certain limitations. First, the specific molecular functions of the genes involved in the constructed lactate metabolism model were not clear. Further research is needed to clarify their expression levels and roles in lung adenocarcinoma. Additionally, more clinical practice data are needed to verify the predictive value of this risk score model in the real-world setting.

## Conclusion

In conclusion, our novel gene signature based on genes related to lactate metabolism has been proven effective in predicting the prognosis and immunotherapy outcomes of patients with lung adenocarcinoma. Our model provides a practical reference for improving the prediction of the efficacy of individualized immunotherapies and guiding clinical practice.

## Supplementary Information

**Additional file 1: Table S1.** 26_lactate_metabolism_related_genes.

**Additional file 2: Fig. S1.** The correlation between the lactate metabolism-related gene signatures and GSVA scores of 50 cancer hallmark pathways.

**Additional file 3:** Raw documents and tables in the process of data analysis.

## Data Availability

The bioinformatics analysis data utilized in this study are publicly available and listed below: the GEO database (https://www.ncbi.nlm.nih.gov/geo/); TCGA (https://cancergenome.nih.gov). And the clinical and prognostic data generated and analyzed during the current study are available in supplementary materials or from the corresponding author on reasonable request.

## Abbreviations

TME: Tumor microenvironment
TCGA: The cancer genome atlas
GEO: Gene expression omnibus
LDH: Lactate dehydrogenase
PDH: Pyruvate dehydrogenase kinase
HAGH: Hydroxyacyl glutathione hydrolase
Tregs: T lymphocytes
IFN-γ: Interferon γ
TAMs: Tumor-associated macrophages
VEGF: Vascular endothelial growth factor
FGF: Fibroblast growth factor
DEGs: Differentially expressed genes
FDR: False discovery rate
GSVA: Gene set variation analysis
LASSO: Least absolute shrinkage and selection operator
HR: Hazard ratios
CI: Confidence interval
CMVs: Copy number variations
CR: Complete response
PR: Partial response
SD: Stable disease
PD: Progressive disease
ORR: Overall response rate
PFS: Progression-free survival
OS: Overall survival
ROC: Receiver operating characteristic
AUC: Area under the curve
RMSE: Root mean square error

## Code availability

All code supporting the current study is deposited in GitHub (https://github.com/xyouli/DNA-based-predictors-of-non-genetic-cancer-phenotypes). All computational analyses are done using public R packages.
