# Supplementary figures and images for: Tumor microenvironment-associated lactate metabolism regulates the prognosis and precise checkpoint immunotherapy outcomes of patients with lung adenocarcinoma
Source: Eur J Med Res. 2022 Nov 21;27:256. doi: 10.1186/s40001-022-00895-6 (PMC9677690; doi:10.1186/s40001-022-00895-6)

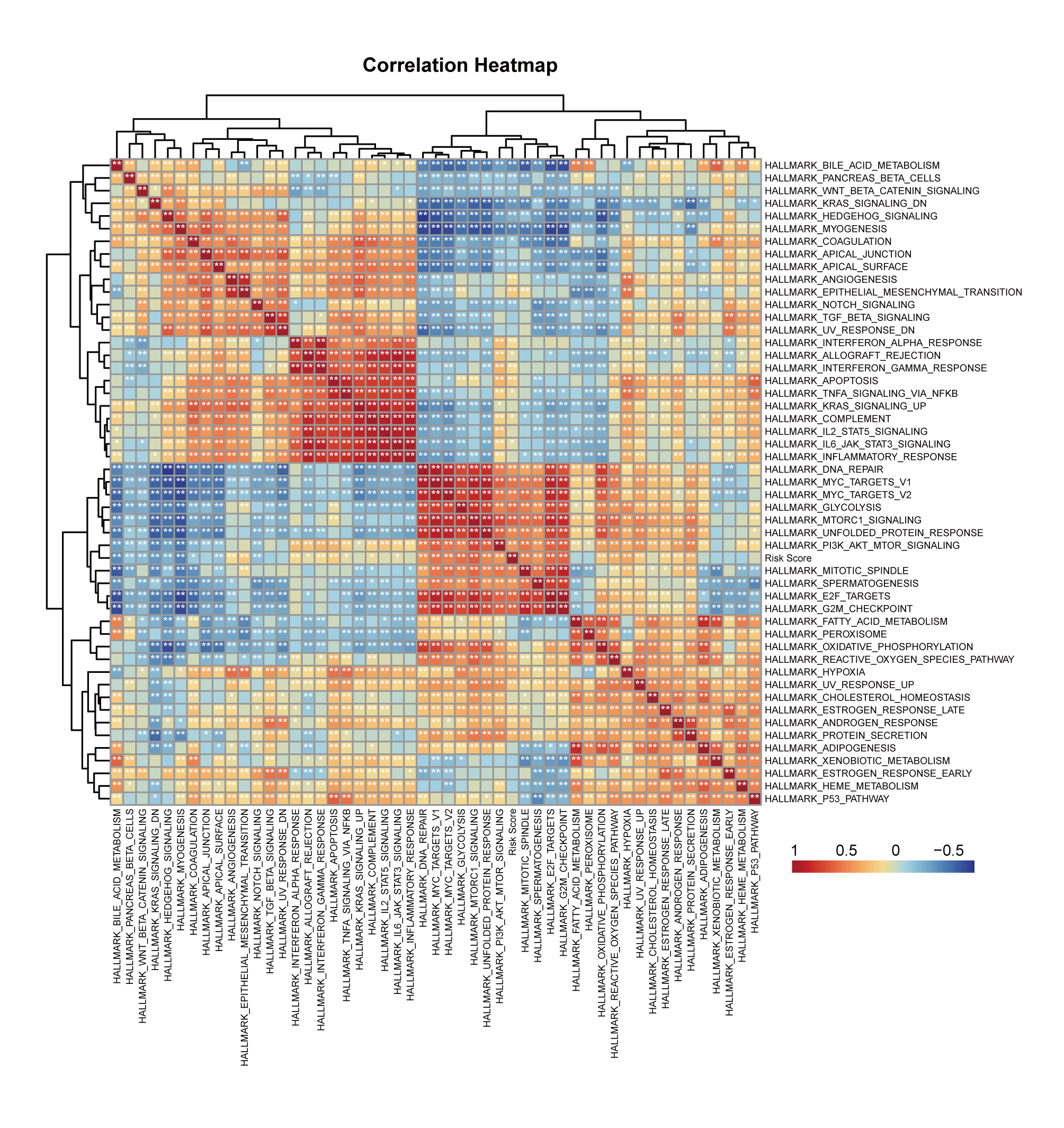

Supplement: Supplementary file 2 — Additional file 2: Fig. S1. The correlation between the lactate metabolism-related gene signatures and GSVA scores of 50 cancer hallmark pathways. [file 40001_2022_895_MOESM2_ESM.tif]
